# Study on the Association among Mycotoxins and other Variables in Children with Autism

**DOI:** 10.3390/toxins9070203

**Published:** 2017-06-29

**Authors:** Barbara De Santis, Maria Elisabetta Raggi, Giorgio Moretti, Francesco Facchiano, Alessandra Mezzelani, Laura Villa, Arianna Bonfanti, Alessandra Campioni, Stefania Rossi, Serena Camposeo, Sabina Soricelli, Gabriele Moracci, Francesca Debegnach, Emanuela Gregori, Francesca Ciceri, Luciano Milanesi, Anna Marabotti, Carlo Brera

**Affiliations:** 1 GMO and Mycotoxin Unit, Department of Veterinary Public Health and Food Safety, Italian National Institute for Health, Viale Regina Elena, 299-00161 Roma, Italy; giorgio.moretti87@gmail.com (G.M.); alessandra.campioni@hotmail.it (A.C.); sabina.soricelli@gmail.com (S.S.); gabriele.moracci@iss.it (G.M.); francesca.debegnach@iss.it (F.D.); emanuela.gregori@iss.it (E.G.); carlo.brera@iss.it (C.B.); 2 Scientific Institute, IRCCS Eugenio Medea, Bosisio Parini, Via Don Luigi Monza, 20-23842 Bosisio Parini, Lecco, Italy; mariaelisabetta.raggi@bp.lnf.it (M.E.R.); laura.villa@bp.lnf.it (L.V.); arianna.bonfanti@bp.lnf.it (A.B.); francescaciceri@libero.it (F.C.); 3 Department of Oncology and Molecular Medicine, Italian National Institute for Health, viale Regina Elena, 299-00161 Roma, Italy; francesco.facchiano@iss.it (F.F.); stefania.rossi.2103@gmail.com (S.R.); 4 National Council of Research, Institute of Biomedical Technologies, Via F.lli Cervi 93, 20090 Segrate, Milano, Italy; alessandra.mezzelani@itb.cnr.it (A.M.); luciano.milanesi@itb.cnr.it (L.M.); 5 Scientific Institute, IRCSS Eugenio Medea, 72100 Brindisi, Italy; serena.camposeo@libero.it; 6 Department of Chemistry and Biology “A. Zambelli”, University of Salerno, Via Giovanni Paolo II, 132, 84084 Fisciano, Salerno, Italy

**Keywords:** autism syndrome, mycotoxins, environment, exposure, IgG, cytokine/chemokine

## Abstract

Environmental factors and genetic susceptibility are implicated in the increased risk of autism spectrum disorder (ASD). Mycotoxins are agricultural contaminants of fungal origin that represent real risk factors for human health and especially for children. Thus, the main hypothesis of this work is that the deterioration of the clinical manifestation of autism in children may result from the exposure to mycotoxins through the consumption of contaminated food. Within a cross-sectional study, a group of autistic children (*n* = 172) and a group of controls (*n* = 61) (siblings and non-parental) were recruited in North and South Italy. All children had blood and urine samples taken, for testing some mycotoxins by a LC–MS/MS validated method. Blood samples were also tested for assessing specific IgG against food and fungal antigens and cytokines. The analyses outputs highlighted statistically significant differences comparing mycotoxins levels between (i) children groups both in urine (deoxynivalenol and de-epoxydeoxynivalenol, *p* = 0.0141 and *p* = 0.0259, respectively) and serum (aflatoxin M1, ochratoxin A and fumonisin B1, *p* = 0.0072, *p* = 0.0141 and *p* = 0.0061, respectively); (ii) a group of selected fungal IgGs, and IgGs against wheat and gluten and (iii) cytokines. These results suggest the need for a deeper examination of the role that mycotoxins may have on the etiology of ASD.

## 1. Introduction

Mycotoxins are agricultural contaminants of fungal origin that may occur in almost all types of crops involving the whole agri-food chain. Indeed, because of their extreme heat stability, mycotoxins are present in untreated seeds up to processed foods and feed as well.

Mycotoxin-producing molds are able to grow at any latitude worldwide and, in the last few years, have been creating a real burden in agriculture that was estimated by the Food and Agriculture Organization (FAO) as 25% of the world’s crops being affected each year. The main consequences for the agri-food chain and human and animal health entail direct implications from an economical point of view, with worldwide annual losses of around 1 billion metric tons of foodstuff and food products and for health-related issues such as the incidence of various diseases, like cancer, gastrointestinal (GI) and neurological impairment [1].

Mycotoxins occur in plant and animal origin products under modified forms or as a cocktail of compounds possibly increasing the effect of their toxicity due to synergistic and/or additive effects.

Due to their chemical structure, mycotoxins have different target organs with a wide range of effects, such as intestinal permeability, oxidative stress, inflammation and fibrosis in some target organs leading to nephrotoxicity, hepatotoxicity, neurotoxicity and immunotoxicity [2,3]. 

Epidemiological evidences are reported in literature where aflatoxins (AFs) have been extensively associated with liver cancer, hepatitis B, morbidity in children suffering from kwashiorkor (a severe protein–energy malnutrition syndrome), stunting and even death in extremely acute exposures [4]. Other mycotoxins were associated to various outbreaks as well: deoxynivalenol (DON) strongly correlates to GI disturbs and fumonisins (FBs) are related to esophageal cancer and neural tube defects. Zearalenone (ZEA) results to be involved in thelarche and gliotoxin (GLIO) is possibly implicated in the etiology of immunologic deficiency. Ochratoxin A (OTA), classified by the International Agency for Research on Cancer (IARC) in Group 2b, i.e., possibly carcinogenic for humans, is suspected to be weakly mutagenic, maybe through oxidative DNA damage, able to exert a causal effect on nephropathies (Balkan Endemic Nephropathy) as well as to increase the incidence of transitional cell (urothelial) urinary cancers [5]. Lastly, it has also been reviewed that AFs, DON and FBs are all involved in intestinal damage through inhibition of protein synthesis (AFs and DON), an increase in systemic proinflammatory cytokines (DON) and inhibition of ceramide synthase (FBs) [6]. The development and the use of biomonitoring helped in food safety to explore exposure scenarios over more traditional approaches. Up to now, as for mycotoxin concern, a number of studies have already been published reporting the scientific evidence of the validation of aflatoxin M1 (AFM1), OTA, DON and FBs as biomarkers of exposure [2,7,8,9,10,11].

The term “autism spectrum disorder” (ASD) is generally referred to a group of neurodevelopmental disorders characterized by a broader phenotype, including typical features such as stereotyped or repetitive behaviours, impaired social activities, and restricted verbal and non-verbal communication [12]. The symptoms manifest generally by 3 years of age, and there is the evidence that often ASD children experience a loss of skills between 18 and 21 months of age, on average, after a relatively normal development [13]. Autism affects more males than females: recent epidemiological studies suggest that the ratio in the prevalence/incidence of ASD is in the range of 2–5:1 male:female [14]. 

The median of prevalence estimates of ASD was reported to be 62/10,000 in a systematic review of epidemiological surveys of autistic disorder worldwide. The prevalence is continuously increasing over time, and this phenomenon can be partially related to the improvement of the diagnostic criteria, switching from other developmental disabilities to ASD [15]. Genetic factors are considered to play an important role in ASD etiology and over 700 genetic loci are implicated in ASD to date. However, most of these are either rare variants, or are common variants in the general population conferring only a small risk for ASD. Additionally, the genomic architecture of ASD is heterogeneous and complex, and it is very difficult to infer common molecular mechanisms. For all these reasons, it has long been hypothesized that gene–environment interaction could be involved in ASD pathogenesis. Among environmental agents, air pollutants, heavy metals, organic toxicants, pesticides, phthalates have long been investigated, as well as seasonal, social and familial environmental factors [16].

Often, ASD is associated to comorbid situation such as cognitive delays, epilepsy, oxidative stress, GI disorders and inflammation. Recently, more attention has been paid on nutritional factors, considering the increasingly important role of gut microbiota and of the so-called “microbiota–gut–brain axis” in determining the human health and the correct development of the central nervous system (CNS) [17]. Gut microbiota, indeed, regulates metabolism and homeostasis and plays a key role in controlling the CNS functions through neural, endocrine and immune pathways [18]. Thus, gut dysbiosis (microbial imbalance) not only leads to inflammatory bowel diseases but also to systemic inflammation altering the CNS functions as described in some neuropsychiatric conditions, including ASD. Some studies have already been conducted to define the bacterial gut composition of ASD patients although with discordant results. Very recently, Strati and collaborators [19] analyzed, for the first time, both the gut microbioma and mycobioma (fungal profile) of ASD children and healthy controls. Interestingly, they found alterations in both the bacterial and fungal kingdom. Specifically, as for prokaryotes, they confirm that ASD subjects displayed a reduction of *Bacteroidetes*, *Dialister* and *Veillonella* as well as an increase of *Lactobacillus*. Regarding the fungal composition, a relative abundance (more than double) of *Candida* was detected in ASD patients *versus* controls revealing that also fungi participate in ASD dysbiosis and providing a new therapeutic target.

Moreover, the observation that often in ASD patients there are recurrent GI abnormalities with a correlation with the severity of autistic symptoms [20] has led to the development of some diets, such as the gluten-free/casein-free diet to reduce or abolish these problems. Interestingly, several studies pointed out that this diet could induce positive cognitive and behavioral effects on ASD children with GI disorders [21], although further data are needed to fully understand the possible mechanisms of action for this diet.

The overlap of the toxicological effects of some mycotoxins with several autistic features e.g., oxidative stress, leaky gut and inflammation that, in turn, impair the microbiota–gut–brain axis, is remarkable. In this scenario, here the presence of Aflatoxin B1 (AFB1), aflatoxin M1 (AFM1), DON, de-epoxy deoxynivalenol-1 (DOM1), fumonisin B1 (FB1), GLIO, OTA and ZEA were has been quantitatively analysed by Ultra-Performance Liquid Chromatography [UPLC^®^]–MS/MS in urine and serum samples collected in a sizable set of ASD patients and controls. The levels found have been associated with measured variables such as a group of IgGs against fungal and food antigens and a group of cytokines/chemokines of pro-inflammatory signals and in immunity responses. This led us to speculate that mycotoxins exposure could have detrimental consequences and participate in the aggravation of medical comorbidities in autistic children.

## 2. Results

### 2.1. Sample Selection

The study enrolled a grand total of 233 children, split into autistic group (*n* = 172), a group of sisters or brothers of the autistics (siblings, *n* = 36) and a group of non-parental children (non-parental group, *n* = 25). In Table 1, the depiction of the group in terms of gender and age is detailed: 131 170 (81.772.9%) males; 29 63 (18.327.1%) females; age ranging between 2 and 12 years. As expected, boys were overrepresented in the ASD case group with a male/female ratio of 4.5/1.

A questionnaire was submitted and responded by the parents or tutors of each child, in order to collect information about clinical issues in the past, with a particular focus on GI alterations and dietary habits. In Table 2, the statistical analyses that gave significant *p* values (≤0.050) are reported.

From the list in Table 2, it is shown that children with ASD are approximately two to eight times more likely to report frequent vomit, diarrhea, abdominal bloating, aerophagia and intestinal anomalies (*p* ≤ 0.050, in bold in Table 2) than the control group. 

### 2.2. Mycotoxins Analysis

Aflatoxin B1, M1, DON, DOM1, FB1, GLIO, OTA and ZEA were analysed by UPLC–MS/MS in urine and serum samples collected from the subjects. For this purpose, a method was developed and validated following international standards [22] and assessing the apparent recovery (R_A_), the signal suppression/enhancement (SSE) and the recovery for extraction (R_E_) [23].

#### 2.2.1. Method Validation

In Table 3, a summary of the method performances obtained during the validation phase is reported. For both biological matrices (urine and serum), the limit of quantification (LoQ), the relative standard deviation (RSDr) and R_A_ of the method, the SSE and the R_E_ are shown.

Apparent recoveries values and matrix effects on signal (measured by SSEs) strongly varied depending on the analyte/matrix combination. The trend of SSE was comparable in both matrices. The strongest suppression was registered by DOM1 both in urine and serum. However, except for GLIO in serum and AFB1 in urine, all the other toxins gave suppression: important in the case of DON and DOM1, modest in all the other cases. As for apparent recoveries, urine gave general best performances in comparison with serum. The percentage values of RSDr, referred to the repeatability of spiked samples, ranged from 10 to 23%.

#### 2.2.2. Occurrence

In Table 4 and Table 5, the description of the dataset (number of observations, percentage values, average, standard deviation, range (minimum–maximum values registered) and median) both as a whole and for each single group (autistic, siblings and non-parental) for the analysed mycotoxins, is reported. 

**Urine.** Excepting for DOM1 and FB1, the percentages of samples with values above LoQ were more than 50% with a peak of 76% for OTA. Excepting for GLIO in all groups and DOM1 in non-parental, all mean values were below the LoQ. Scrutinizing the co-occurrence of the toxins in urine in the whole dataset, 3% of the samples (*n* = 5) showed positive values (≥LoQ) for the eight mycotoxins, while 32% (*n* = 52) showed the presence of AFB1, AFM1 and OTA, and 9% (*n* = 14) the presence of DON, FB1 and ZEA. DOM1 showed the lowest number of positive results (28.4% in the whole group, 34.6% in the autistic group, 8% in siblings and 22.7% in the non-parental group).

**Serum.** Excepting for OTA levels in all groups and AFM1 in whole and autistic group, the percentages of positive samples were below 50%, the highest percentage being for OTA in autistic children (85%). Apart from OTA in all groups, all mean values were below the LoQ. Scrutinizing the co-occurrence of the toxins in urine in the whole dataset, no sample was positive for the eight mycotoxins, while 4% (*n* = 9) showed the co-presence of AFB1, AFM1 and OTA and 2% (*n* = 5) the presence of GLIO, AFB1, AFM1 and OTA. ZEA showed the lowest number of positive results (5.4% in the whole group, 5.2% in the autistic group, 8.2% in siblings and 0 in the non-parental group).

#### 2.2.3. Statistical Analysis

The hypothesis of normality distribution (Shapiro–Wilk test) was refused, thus non-parametrical tests (which do not imply any distribution assumption) were used for the statistical treatment. The possible differences between concentration levels of mycotoxins in autistic children and control groups (either considered as a unique control or split into sibling and non-parental groups) were explored by a Wilcoxon rank-sum test.

Sub-groups with specific co-morbidity were also clustered as described in Table 6.

Finally, two more sub-groups were defined: (1) ASD paired sub-group (sub-group of autistic children with one brother/sister in the control group) and (2) sibling paired sub-group (sub-group of siblings of autistic children). For the statistical analysis, taking into consideration the dependence, the Wilcoxon signed-rank test was applied.

In Table 7, all differences between autistic children and controls or sub-groups of autistic children that showed a *p* ≤ 0.050 for mycotoxin content in urine (_u) or serum (_s) are summarised.

**Urine.** DON and DOM1 levels in autistic children showed mean values statistically different when compared with levels of the control group considered as a whole (sibling and non-parental together) (*p* = 0.014 and *p* = 0.0259, respectively); likewise, mean levels of DON and DOM1 were statistically different when compared with siblings (*p* = 0.0185 and *p* = 0.259, respectively).

When GLIO levels in the autistic group were stratified for age, a statistical difference was found (*p* = 0.0127).

By dividing the autistic children group in SG1+ and SG1−, the following differences were found: OTA levels were statistically different for *Candida albicans* (*p* = 0.0169), *Fusarium moniliforme* (*p* = 0.0075); AFB1 for *C. albicans* (*p* = 0.0462); DOM1 for *C. albicans* (*p* = 0.0487) and FB1 for *C. albicans* (*p* = 0.0126).

The same analyses of the differences were applied to the subgroup of autistic children with a subgroup of brothers or sisters in the sibling group. Statistical significance was found in the difference of DON levels (*p* = 0.0305) and OTA levels (*p* = 0.0272).

To assess the relationship between mycotoxin level in urine and the variables, a Spearman’s rank correlation coefficient (ρs) was calculated. Negative and positive correlation was found and the following couples with *p*-values < 0.050 were considered as a significant result: GLIO and *A. flavus* (ρs = −0.2631 *p* = 0.0096); FB1 and *A. clavatus* (ρs = −0.2046 *p* = 0.0455); AFB1 and wheat (ρs = 0.2188 *p* = 0.0322); AFB1 and gluten (ρs = 0.2485 *p* = 0.0061); DON and *A. fumigatus* (ρs = −0.2777 *p* = 0.0061).

**Serum.** OTA and AFM1 levels in autistic children showed mean values statistically different when compared with control group considered as a whole (sibling and non-parental together) (*p* = 0.0141 and *p* = 0.0072, respectively); differences were also found when they were compared with sibling control group (non-paired) (*p* = 0.0261 and *p* = 0.0262, respectively).

When mycotoxin levels in the autistic group were stratified for age, OTA and DOM1 showed a statistical difference (*p* = 0.0376 and *p* = 0.0362, respectively).

By dividing the autistic children group for co-morbidity, SG1+ and SG1−, the following differences were found: GLIO levels were statistically different for *A. flavus* (*p* = 0.0169); OTA for wheat (*p* = 0.0212); AFM1 for *A. nidulans* (*p* = 0.0457).

Differences of AFM1 mean levels resulted statistically different (*p* = 0.0385) also in SG4+ and SG4−.

The analyses of the differences were performed to a subgroup of autistic children, which had a brother and/or sister in the sibling group and with a subgroup of brothers or sisters (by applying a Wilcoxon signed-rank test). Statistical significance was found for OTA (*p* = 0.0409).

To assess the relationship between mycotoxin level in urine and serum and the variables, a Spearman’s rank correlation coefficient was calculated. Negative and positive correlation was found as reported in Table 8, where the ρs obtained with *p*-values ≤ 0.050 are reported. GLIO and *A. flavus* (ρs = 0.2258 *p* = 0.0270); OTA and *A. fumigatus* (ρs = −0.2466 *p* = 0.0154); AFB1 and *A. oryzae* (ρs = −0.2232 *p* = 0.0288); AFB1 and *A. clavatus* (ρs = −0.2230 *p* = 0.0290).

### 2.3. Clinical Parameters and Cytokines Analyses

The quantitative analysis of several serum cytokines and chemokines indicated some significant differences between healthy control and autistic children. Therefore, the statistical comparison was carried out and results have been summarized in Table 9. All correlations are positive and values of Spearman’s rank correlation coefficient result from weak to moderate.

## 3. Discussion

### 3.1. Occurrence

This study presents a multi-mycotoxin method suitable to detect a group of mycotoxins in urine and serum. Some R_A_ in serum was below 60% with high suppression signal but, considering the precision data values (≤23%), these results were considered reliable, especially considering the advantage of a multi-mycotoxin detection (see Table 3).

Taking into consideration the data as a whole, the present work constitutes a densely populated dataset and a precious source of information about possible biomarkers of exposure to mycotoxins in Italian children. 

OTA is the mycotoxin with higher numbers of positive results both in urine and serum (74.8% and 82.9% of positive samples, respectively), followed by GLIO (71.6%), AFB1 (66.2%), AFM1 (62.6%) in urine samples and by AFM1 (50.2%) and AFB1 (22.9%) in serum samples.

As for levels of OTA, DON, AFB1 and AFM1 found in urine, these are in line with other data in literature for children or adults [24,25,26].

More specifically, OTA mean values found in this study in autistic children were in agreement with the mean values found in a pilot study that was conducted in a smaller autistic children group (*n* = 52) and controls (*n* = 58) [27] (0.06 ng/mL in urine and 0.40 ng/mL in serum). OTA positivity seems to be of high importance, since it contaminates most of food products especially those consumed by children, such as cereal-derived products and chocolate.

Regarding DON and DOM1, our results are slightly different from those presented in the experimental study of DON biomarkers in urine performed by the European Food Safety Authority (EFSA) [28] to assess the exposure to DON in the European Union (EU) population. In that study, the Italian group of children and adolescents showed almost 100% of subjects positive to DON (among 80 to 100%, 0.43–75.9 ng/mL range, mean) and 0% of positive results to DOM1 for the same group. Here, considering DON, about 40% of urine samples were above the LoQ with mean value only of 1.4 ng/mL, while DOM1 resulted positive on the 27% of the samples with a mean value of 8.4 ng/mL.

AFB1 and AFM1 showed a substantial number of positive results both in urine and serum. Considering their toxicity (AFB1 is carcinogenic and genotoxic, classified in group 1 by IARC [29]) this constitutes a concern of exposure.

GLIO values found in the present study confirmed percentages values of positive results higher in urine than in serum, though mean levels were slightly lower than those of the pilot study [27]. 

FB1 was tested in urine and serum, resulting positive in a small number of samples (44% and 13.7% in urine samples and in serum samples, respectively) with low levels, very close to LoQ values.

As regards the exposure to ZEA, it was undetectable in 50% of urines. Conversely on what was found in [27], where ZEA, α-ZEA and β-ZEA were all not detected, positive values for ZEA in urine were found close to LoQ (range 1.6–6.5 ng/mL). The lower LoQ values (1.6 ng/mL in the present method vs. 5 ng/mL in De Santis et al. [27]) have increased the detectability of positive results, when present. This also explains the higher number of positive results found in the present study in comparison with the ones published by Duringer [30]. In fact, Duringer conducted a screening for urinary mycotoxins in 25 autistic patients and 29 healthy controls and found one positive result for 3-Acetyldeoxynivalenol. Indeed, they tested 87 urinary mycotoxins via liquid chromatography–tandem mass spectrometry, presenting a good screening. To note, as they also highlighted, they used a dilute and shoot sample preparation with high LoQs (almost 10 times higher in comparison with those reported here), thus, explaining the reported differences. 

### 3.2. Statistical Associations

As regards the statistical analyses that were conducted, we focused on the comparison between the patients group and the control group to find out possible differences and associations between mycotoxins and other variables in ASD. In this respect, we excluded comparisons with those mycotoxins that had less than 15% of positive results (namely, ZEA and DOM1 and FB1 in serum samples) in order to avoid misreported outputs because of the number of zeros or values below LoQs.

Statistically significant differences are shown in Table 7. These differences are not intended to explain the pathobiology of the disorder, but rather to give information on the presence of possible environmental risk factors that may act as perturbative agents involved in the gene–environment interaction, the more accredited hypothesis for ASD onset.

Differences of OTA, AFM1 levels in serum and DON and DOM1 levels in urine were found between autistics and the control group (siblings and non-parental, summed) and (excepting DOM1) between autistic and siblings group. Furthermore, OTA and DON in urine confirmed statistically significant differences when autistic children were paired and compared with their brother or sister. 

These differences in the levels of exposure in subjects from the same family that presumably have the same diet, should be deeply explored as the same toxins can have effects of different extent depending on individual predisposition. According to Smith [6], the intestine is a major target for the toxic effects of AFs, DON and FBs, because of their effect in the impairment on nutrient uptakes and intestinal pathology. Altered intestinal architecture can result in a loss of enzymes, leading to malabsorption of a variety of nutrients. AFs, DON and FBs may therefore share a convergent pathway in which mucosal damage can lead to impaired nutrient absorption and/or increased intestinal permeability [6]. Thus, to be clarified is the perturbative effect of chronic exposure to these toxins by ASD children with GI issues, which can favour or worsen persistent damages, and/or with microbioma–brain–gut signalling that may play a role in brain development or result in neuroplastic and functional alterations [31].

To explore the relationship between food- or mould-specific IgGs levels of ASD patients and mycotoxins, two subgroups were defined: one with IgGs levels >20 mg/L and one with IgG values ≤20 mg/L. Some mycotoxins highlighted differences that were statistically significant especially among specific fungal IgG groups as reported in Table 7.

SG1+ and SG1− were defined for food-specific IgGs and the only difference regarded OTA levels in serum in SG1+ for wheat group.

Correlations, by assessing Spearman’s rho, were run to determine relationship between couples of mycotoxins and IgGs values in the autistic group (Table 8). Weak, positive and negative monotonic correlations were determined: *A. clavatus* and AFB1 and FB1; *A. flavus* and GLIO; *A. fumigatus* and OTA and FB1; *A. niger* and DOM1; *A. orizae* and AFB1; gluten and AFB1; wheat and AFB1; β-lactoglobulin and DOM1.

GI issues and associated symptoms are commonly reported in individuals with ASD [32], and from large prospective well-controlled studies it was confirmed that GI problems, particularly constipation and diarrhoea, affect children with ASD and developmental delay far more often than children with typical development [33]. Our results also evidenced that recurrent vomit, aerophagia, diarrhoea and intestinal anomalies are more likely to occur in autistic children.

It is thus plausible to hypothesize that the toxic effect of mycotoxins as well as their biological consequences may contribute to the aggravation of the general conditions of patients including leaky gut for which the increased permeability, hypothesizing barrier defects and affecting the mucosal immune system, lead to changes in gut flora [34].

This association among mycotoxins and autism was explored by a study of Duringer and co-workers [30]. Due to the low number of positive results (equal in both groups), they could not find any kind of difference or correlation between ASD and the control group. Indeed, their study has a smaller sample size (25 autistic children and 29 children as controls), a lack of sensitivity for the sample preparation (LoQ for deoxynivalenol was 50 µg/L) and no genetic test was previously performed on patients so the inclusion of subjects with causative genetic variations cannot be excluded.

The statistical differences of serum levels of cytokines reported in Table 9 indicated interesting and significant correlations between mycotoxin levels and cytokine/chemokines in autistic group. The cytokine/chemokines correlated to mycotoxin levels are known to be involved in several pathological conditions, in particular platelet derived growth factor (PDGF) and tumor necrosis factor (TNF)-alpha are considered important pro-inflammatory signals, IL-13 and eotaxin are involved in inflammation and allergic conditions, as well as IL-7 and IL-12 in immunity responses. Alterations of plasmatic levels of some of the cytokines reported in this study were also described in other studies [35,36]. The cytokine levels differences suggest that exposure to mycotoxins may represent a portion of a complex scenario involving both innate and adaptive immune responses. Due to the reported co-morbidity of other diseases with ASD (just as an example the above reported GI issues) [32], this serum quantification of cytokines confirms a key role of immune system in ASD children pathology. Nevertheless, it is not yet clear whether the pro-inflammatory phenotype observed coupled to the GI symptoms may represent the effect of a more general immune system deregulation or whether they play a role in ASD pathogenesis. Further, it should also be taken into account that significant alterations of microbiota (including bacteria and fungi) and GI mucosa in ASD children (strongly suggested by occurrence of diarrhoea and intestinal anomalies) might facilitate the entry of toxins and/or antigens into the blood stream, as well as mycotoxins, and may mediate intestinal damage [6]. Several pathogenic bacteria strains, indeed, produce lipopolysaccharides that increase the intestinal tight junction permeability [37] as well as some other produce pore-forming toxins, protein exotoxins that induce pores formation in the cell membrane [38]. The increase of *Candida* genus, just found by Strati and collaborators in the gut of ASD children [19] further support the role of “leaky gut” in xenobiotic adsorption and consequent immune reactivity. Indeed, in animal model, *Candida* colonization of intestinal tract promotes the absorption and sensitization against food antigens [39]. Moreover, gut eubiosis (beneficial microbes) produces short chain fatty acids (SCFAs) that, in turn, increase the production of the tight junction proteins of brain-blood barrier (BBB). On the contrary, dysbiosis decreases SCFAs production that affects the integrity of BBB [18] and BBB permeability has already been described in ASD patients [40].

### 3.3. Interventions

Intestinal permeability plays a key role in food allergies and intolerances and in autistic children we have found a correlation between exposure to OTA, ZEA, AFB1, DOM1 regarding wheat, and gluten. The gluten-free/casein-free diet is a recurring finding in the literature with regard to autistic children, with alternate results. The tendency of ASD children to eat often the same foods can cause an overload in the intake of several substances and contaminants, such as mycotoxins. Also interesting is the detection of IgGs against fungal strains that denounce the contact with the fungus. We believe that in ASD children with GI problems and altered intestinal permeability, intolerances should be sought and, if positive, a rotation diet should be applied [41] together with probiotics, in order to improve their clinical issues. 

## 4. Experimental Section

### 4.1. Materials and Methods

#### 4.1.1. Subject Enrolment: Inclusion Criteria

A group of autistic patients (*n* = 172) and a control group (*n* = 61) were recruited by the Italian Research Hospital, E. Medea (Istituto di Ricovero e Cura a Carattere Scientifico, IRCCS), from two locations: Bosisio Parini (Lecco, Lombardia, Italy) and Ostuni (Brindisi, Puglia, Italy).

During the first contact with children and their families (or tutors), medical staff gave the presentation of the research project detailing context and aims. The medical staff interviewed all those who decided to participate. Children were tested with specific psychological tests; urine and serum samples were collected.

For autistic children, the inclusion criteria were the following: age in the range 2 to 12 years old, diagnosis of autistic spectrum disorder, with exclusion of other genetic syndromes (e.g., X fragile), epilepsy, or other medical disorders or neurological disturbances.

The Ethical Committee of IRCCS Eugenio Medea-La Nostra Famiglia, approved the study.

The autistic diagnosis was verified by a team of psychiatrics and psychologist applying the Autism Diagnostic Observation Scale 2 [42] and The Autism Diagnostic Interview [43]. The same medical team submitted other tests for the assessment of adaptive behaviour (Vineland Adaptive Behaviour Scale) [44], for the measure of the breadth of repetitive behaviour (Repetitive Behavior Scale-Revised) [45] or Griffiths Mental Development Scales [46], for the assessment of intelligence scale (preschool and primary scale Wechsler intelligence scale) [47,48], and for the identification of problem behaviour (the Child Behavior Check List, CBCL) [49,50]. 

The control group was composed of a subgroup of siblings (*n* = 36) and a subgroup of non-parental (*n* = 25), which did not have any parental relationship with the autistic group. The CBCL [49,50] was applied to the control group in order to exclude possible pathologies. 

Besides, medical history and food habits were recorded through *ad hoc* questionnaires. A medical general questionnaire was completed by the parents to acquire info on the general status of the mother (before and during pregnancy) and of the children (from the birth to that moment), on the ingestion of drugs; the presence of GI disorders, of food allergies or intolerances, and special dietary needs in children and adolescents were assessed.

#### 4.1.2. Specimen Collection

All those who decided to participate were informed on how to collect first morning urine samples on the provided sterile tubes. The children blood sampling date and the children urine samples consignment was agreed with the parents. 

For the analyses, an aliquot (50 mL) of urine was delivered, while the blood sample was taken by the medical staff. Biological samples were stored at −20 °C until delivery to the Italian National Institute for Health laboratories (ISS, Rome), where they were stored at −20 °C until analyses.

#### 4.1.3. Mycotoxin Analysis

Analyses of AFB1 and AFM1, OTA, DON, DOM1, ZEA, FB1 and GLIO were performed by GMO and Mycotoxin Unit, Italian National Institute for Health. 

Aflatoxin B1, AFM1, OTA, DON, DOM1, ZEA, FB1 and GLIO were tested in *serum* and urine with method previously validated for precision and trueness performances.

The principle of the methods for the mycotoxin analyses in serum and urine are briefly reported.

Mycotoxins from *serum*: the extraction procedure was performed on 1 mL of sample to which 18 μL of pronase (Sigma-Aldrich, Saint Louis, MO, USA) and 1 mL of phosphate buffer were added prior to the incubation step (overnight, 37 °C). The sample was mixed and vortexed with 2 mL of ethyl acetate 1% formic acid, vigorously shaken in a laboratory shaker for 30 min and then centrifuged 15 min at 4000 rpm. The organic phase was transferred to a clean vial and gently dried under a gentle N_2_ stream. After adding and mixing the aqueous phase with 1 mL of acetonitrile, 1.6 g of QuEChERS (DisQUE, Waters, Milford, MA, USA) were also added. The sample, vigorously mixed (3 min vortex), was then centrifuged 15 minutes at 4000 rpm. The recovered supernatant was added to the same clean vial and gently dried under a gentle N_2_ stream.

Mycotoxins from *urine*: the extraction procedure was performed on 3 mL of sample to which 3 mL of ethyl acetate 1% formic acid was added. The sample was vigorously shaken in a laboratory shaker for 30 min and then centrifuged for 15 min at 4000 rpm. The organic phase was transferred to a clean vial and gently dried under N_2_. After adding and mixing the aqueous phase with 3 mL of acetonitrile, 2.1 g di QuEChERS (DisQUE, Waters, Milford, MA, USA) were also added. The sample was vigorously shaken for 10 min, and then centrifuged for 15 min at 4000 rpm. The recovered supernatant was added to the same clean vial and gently dried under a gentle N_2_ stream.

Both dried serum and urine samples were reconstituted with 0.3 mL of injection solvent (MeOH/H_2_O 3% formic acid and 5 mM ammonium formiate, 50/50, *v*/*v*). The samples were then filtered on 0.22 μm membrane disc filters polyvinylidene difluoride (PVDF) before injection.

The mycotoxins were detected on an Acquity UPLC, Quattro Premier LC–MS/MS system (Waters Corporation, Milford, MA, USA), via electrospray ionization. The chromatographic separation was obtained in UPLC with a binary gradient elution mode, at 40 °C, on a C18 Kinetex column (Biphenil 100 mm, 2.6 μm, 50 × 3 mm; Phenomenex, Torrance, CA, USA) and an in-line filter unit.

Both eluent A and eluent B, composing the mobile phases, contained 5 mM ammonium formiate and 3% formic acid and were made up of acetonitrile/water 50/50 (*v*/*v*; eluent A) and 50/50 methanol/water (*v*/*v*; eluent B). After an initial time of 2.5 min at 95% eluent A and 5% eluent B, the proportion of B was increased to 25% (75% eluent A) for further 1.5 min, to 60% (40% eluent A) for further 2 min, to 90% (10% eluent A) for further 1.5 min, and to 100% for further 1.5 min. After that, the column was re-equilibrated for 3.5 min at 95% eluent A and 5% eluent B. The flow rate was 0.3 mL/min.

Electro Spray Ionisation (ESI)–MS/MS was performed in multiple reaction monitoring (MRM) mode in positive polarity with the following settings: a capillary voltage of 3.5 kV, a desolvation temperature of 350 °C, a desolvation gas flow of 600 L/h, a cone gas flow of 100 L/h and nebuliser gas fully open, all nitrogen. Dwell time was 0.02 s. The collision gas was argon at 3.5 10^−3^ atm on a pressure regulator. Ionisation and MS/MS collision energy settings were optimised infusing separate mycotoxin solutions (1–2 mg/mL) at a flow-rate of 10 mL/min. For each mycotoxin, the parent and two daughter ions were recorded; the optimized conditions are listed in Table 10. Masslynx and Quanlynx softwares were used for data acquisition and processing.

Primary standards (Biopure by Romer Labs^®^ (Getzersdorf, Austria) and Sigma-Aldrich^®^, Saint Louis, MO, USA) were diluted in appropriate working solutions for the preparation of neat standard calibration curves. Reagents Liquid Chromatography (LC) gradient grade methanol and acetonitrile, as well as MS grade ammonium formiate and formic acid were purchased from Sigma-Aldrich^®^.

#### 4.1.4. Method Validation

The performance characteristics of the method for urine and serum for the seven mycotoxins were obtained with replicated (*n* = 3) spiking experiments carried out at five concentration levels in the relative concentrations of 1:2:3:4:6 in the final diluted extracts. Neat standard calibration curves for all the mycotoxins were prepared by dilution of appropriate amounts of the final working solution with methanol: water 0.3% formic acid, 5 mM ammonium formiate (50/50, *v*/*v*) at levels close to those used in spiked samples. For the assessment of matrix effects and extraction efficiency (recovery), the diluted blank urine and serum extracts were fortified at the concentration range matching the neat standard calibration curve.

For the validation of the method, the whole procedure was carried out in triplicate at different concentration levels: 5 levels for urine (relative concentrations of 1:2:5:14:50) and 9 levels for serum (relative concentration of 1:1.5:3:4.5:6.5:10:15:20:30) were performed. From these levels, the *spiked sample curve* was obtained. The validation protocol also included the testing of blank extracts (performed in triplicate) spiked after extraction at concentration levels comparable with the matrix (urine or serum); from these levels, the *spiked extract curve* was obtained. Moreover, neat standard calibration curves for each analyte were run by injecting 5 levels of analyte (mixed standards with relative concentrations of 1:3:6:15:30).

The apparent recovery or absolute recovery of the method (R_A_), the signal suppression/enhancement (SSE) due to matrix effects and the recoveries as extraction efficiency (R_E_) were evaluated on the basis of the obtained slopes of the 3 curves constructed by plotting the signal intensity versus the analyte concentration [16]. Equations (1)–(3) below describe how SSE, R_A_ and R_E_ were calculated. 

SSE = 100 × SLOPE_SPIKED BLANK CALIB CURVE_/SLOPE_NEAT STANDARD CALIB CURVE_ (1)

R_A_ = 100 × SLOPE_SPIKED CALIB CURVE_/SLOPE_NEAT STANDARD CALIB CURVE_ (2)

R_E_ = 100 × R_A_/SSE (3)

Limits of quantifications (LoQs) were calculated based on signal-to-noise (S/N) ratio of 10/1.

#### 4.1.5. Clinical Parameters and Cytokine Analyses

Measurement of food- and mould-specific allergological IgG (Table 11) were performed in IRCCS E. Medea. Cytokine analyses were performed by the laboratory of the Facility for Complex Protein Mixture (CPM) Analysis at ISS, Rome.

Allergological food- and mould-specific IgG (Table 10) were assessed by Immulite 2000 XPi automated system with an allergen-specific IgG test on a solid-phase, enzyme-labelled chemiluminescent immunometric assay. Chemiluminescent signal was measured as proportioned to the bound enzyme (Standard World Health Organization (WHO) WHO 1st International Reference Preparations (IRP) 67/86). The analytical sensitivity of the assay is 1 mg/L (reportable range of the test: 2–200 mg/L—WHO 1st IRP 67/86). Regarding analytical specificity, the endogenous interference studies showed no significant effect on patient results with the following substance concentrations: bilirubin up to 200 mg/L, hemoglobin up to 522 mg/dL, and triglycerides up to 3000 mg/dL (IMMULITE 2000 systems Allergen Specific IgG instructions, Siemens Healthineers, Malvern, PA, USA). The detection antibody for the assay is a murine monoclonal anti-human IgG antibody that is specific to total human IgG.

Cytokines and growth factors in sera from children affected by ASD and controls were measured by Luminex technology using a Bio-Plex Pro Human Cytokine 27-plex panel, (Bio-Rad Laboratories, Hercules, CA, USA) for the following different analytes (PDGF-BB, Il-1ra, Il-4, Il-5, Il-7, Il-12, Il-13, IFN-g, EOT, TNF-alfa, RANTES). The analysis was performed using 15 µL of the serum as per manufacturer’s instructions. After the incubation with antibodies-activated magnetic beads, samples were washed using a Bio-Plex Pro TM Station (Bio-Rad) as described [51], diluted and incubated with antibodies-activated magnetic beads then washed using a Bio-Plex Pro TM Station (Bio-Rad) according to the manufacturer’s instructions. The quantification was carried out with a Bio-Plex Array Analyzer (X200, Bio-Rad) and analyzed with Bio-Plex Manager Software version 6.1. Results were expressed as pg/mL.

#### 4.1.6. Statistical Analysis and data Handling

Shapiro–Wilk test was used to verify the distribution of the quantitative data. As the hypothesis of normality was refused, non-parametrical (which do not imply any distribution assumption) set of tests were used for the statistical treatment. More specifically, in order to assess the possible differences between concentration levels of mycotoxins in autistic children and control groups, the Wilcoxon rank-sum test (Mann–Whitney two-sample statistic [52,53]) was used. Where the collation of variables implied more than one distribution (Intelligent Quotients (IQs or age groups), Kruskal–Wallis test was used [54].

In order to reduce/control the genetic variability of the syndrome, two groups were defined: (1) a group of autistic children with one brother/sister in the control group and (2) a group of siblings of autistic children. For the statistical analysis, taking into consideration the dependence, the Wilcoxon signed-rank test was applied.

Lastly, to assess the correlation between mycotoxin levels and quantitative variables, a Spearman’s rank correlation coefficient (or Spearman’s rho) was used.

All tests were conducted with a level of significance of 5%. All outputs are reported together with the level of statistical significance, *p*-value. Analyses were conducted by means of STATA14 software (Stata/IC 14.0, Copyright 1985–2015 StataCorp LP).

As regards the data handling for the setting of the data set, depending on the nature (if quantitative or qualitative), variables were treated as follows:

1. Mycotoxins: quantitative, non-negative variable expressed as ng/mL. Values were assigned as follows:
  - Value = 0 ng/mL; when no analytical result derived from the analysis, thus no concentration could be derived;
  - Value = LoQ/2 ng/mL; the half of the limit of quantification (LoQ/2) was assigned to those analytical results that gave a value included between zero and the limit of quantification. These samples could not be quantified with the established precision of the method (below 23%).
  - Values = numerical value to all analytical results ≥LoQ μg/mL; the value is expressed in ng/mL rounded to the second figure after the comma for (DON, DOM1, FB1 and ZEA), and to the third figure after the comma for OTA, AFB1 and AFM1.
2. IgG quantitative, non-negative variable expresses ad mg/L. In addition to the quantity, a stratification was performed generating a binary on the basis of a threshold of 20 mg/L:
  - Value “0” when the IgG value is ≤20 mg/L;
  - Value “1” when the IgG value is >20 mg/L
3. Cytokines: quantitative, non-negative variable: expressed as pg/mL.
4. The variables age, intelligence quotient, GI problems and verbal were stratified as follows:
  - Age. This variable has been set defining the age at the moment of the sampling. The age was split in three classes: 2–4.5 years old; >4.5–7 years old; >7 years old.
  - Gastrointestinal Issues (GI). This is a dummy variable: presence of GI takes the value “1”, when one (or more) of the answers related to GI problems was positive, absence takes the value “0”, when all the answers related to GI problems were negative. It was derived on the basis of the answer given on the questionnaires on the presence or absence of the following: Vomit, Diarrhoea, Constipated, Stomach-aches, Painful bowel, Gastroenteritis, Aerophagia, Abdominal bloating, Lack of appetite, Intestinal infections, Intestinal anomalies.
  - Verbal. This is a dummy variable: presence of verbal language skills takes the value “1”, absence takes the value “0”. The verbal language skill was derived from the tests submitted to the autistic patients.
  - Regressive. This is a dummy variable: presence of regressive pattern takes value “1”, absence takes value “0”. The regressive pattern was derived from the tests submitted to the autistic patients.

## 5. Conclusions

Recent data indicate that in the last few decades there has been an alarming increase in childhood neurobehavioral disorders, for example from learning difficulties, attention deficit disorders, to the most severe forms such as mental retardation and autism, and the syndromes related to it. Such an increase in the last decades is partly due to greater sensitivity and improved diagnostic criteria but there is sufficient evidence to hypothesize a role for a number of environmental risk factors for the general population, and for children in particular, food being recognized as a priority exposure both for natural substances, such as mycotoxins, and other bioactive xenobiotics, such as phytoestrogens [55]. These xenobiotics can be internalized at various levels of the digestive process going from the flora intestinal bacterial, intestinal tissue itself until the liver. The health impact that mycotoxins have is well established and for this reason it is imperative to maintain the level of these toxic substances in food as low as possible. Our results show that ASD patients have a significant amount of several mycotoxins in their body fluids with respect to the controls, even with respect to their siblings that should share the same environment and food habits. 

It is noteworthy that the mycotoxins effects on cell chemotaxis have been reported [56,57,58] as well as chemokine pathways and cell acidification involvement in endothelial and neurodevelopmental disorders [59,60,61] have been demonstrated. Our data reported support these findings and open new interesting perspectives connecting mycotoxins, chemokines and neurodevelopmental disorders. 

Heterogeneous and variable phenotypes in autistic syndromes are framed in a *gene- environment-immune system* complex grid and, even if any of the possible interrelationships into the triad has been elucidate yet, the multifactorial etiology is the only key that may explain this complexity [34]. This suggests that ASD patients exposed to the negative effects of these known xenobiotics can experience detrimental consequences, proposing mycotoxins as triggering factors for the impairment of medical co-morbidities in autistic children.

The association of mycotoxins with IGgs and cytokine/chemokines elicit to consider these xenobiotics among the perturbative agents involved in the gene–environment interaction in the attempt to elucidate the role of environmental risk factors in the pathogenesis for ASD onset.

This study opens the way to environmental xenobiotics to launch further relevant investigations that we intend to pursue to investigate the association between negative effects of mycotoxins on child health and ASD susceptibility.

## Figures and Tables

**Table 1:** Demographic and clinical characteristics of the subjects.

	Autistics	Siblings	Non-Parental
N of subjects (233 in total)	172	36	25
Sex (Male)	81.9%	41.6%	56%
Male/Female ratio	141/31	15/21	14/11
Age (mean ± SD *)	5.68 ± 2.58	7.95 ± 3.84	8.53 ± 2.03
New-born length (cm)	49.16 ± 5.44	50.20 ± 2,44	50.02 ± 1,87
New-born weight (gr)	3181.72 ± 705.81	3293.09 ± 450.04	3297.73 ± 473.75
Head circumference (cm)	34.55 ± 2.83	34.34 ± 1.87	34.34 ± 1.87	34.08 ± 1.40
Regressive pattern	30.1%	-	-
Location (referred to Nord)	72.9%	66.7%	87.05%
Non-verbal	58 (33.7%)	-	-
Gastrointestinal problems (referred to *N* = 233)	26.6%	2.6%	2.1%

* Standard deviation.

**Table 2:** Results of the gastrointestinal problems between ASD group and control group (siblings and non-parental, summed).

Gastrointestinal Problems	Autistics	Control (All)	Odds Ratio	*p*-Value
Vomit	65	18	4.28	**0.024**
Diarrhoea	83	26	2.41	**0.050**
Constipated	85	24	1.92	0.169
Stomach-aches	48	16	1.74	0.620
Painful bowel	60	23	0.65	0.389
Gastroenteritis	58	25	0.96	0.940
Aerophagia	60	16	8.07	**0.024**
Abdominal bloating	56	21	1.74	0.313
Lack of appetite	56	19	3.02	0.068
Intestinal infections	171	60	1.82	0.079
Intestinal anomalies	169	60	2.81	**0.007**

**Table 3:** Method performances in urine and serum.

	LoQ ^a^, ng/mL	R_A_^b^, %	SSE ^c^, %	R_E_^d^, %	RSDr ^e^, %	LoQ, ng/mL	R_A_, %	SSE, %	R_E_, %	RSDr, %
Compound	Urine					Serum				
GLIO ^f^	1.1	97	86	112	22	11	63	104	61	15
OTA ^g^	0.16	69	76	90	17	0.16	56	79	71	21
AFB1 ^h^	0.03	103	111	93	23	0.01	82	92	89	22
AFM1 ^i^	0.32	70	71	99	18	0.22	52	72	73	19
ZEA ^j^	1.6	78	82	95	19	1.0	50	87	57	19
DON ^k^	1.4	66	73	90	10	5.0	59	62	97	13
DOM1 ^l^	5.0	67	58	115	23	5.0	60	54	111	14
FB1 ^m^	1.1	72	64	112	10	3.0	56	85	66	23

^a^ Limit of Quantification; ^b^ Apparent recovery; ^c^ Signal Suppression/Enhancement; ^d^ Recovery for extraction; ^e^ Relative Standard Deviation (repeatability); ^f^ Gliotoxin; ^g^ Ochratoxin A; ^h^ Aflatoxin B1; ^i^ Aflatoxin M1; ^j^ Zearalenone; ^k^ Deoxynivalenol; ^l^ De-epoxy deoxynivalenol-1; ^m^ Fumonisin B1.

**Table 4:** Description of the whole dataset for the mycotoxins analysed in urine samples.

Group	Observations	% Positive	Mean (ng/mL)	SD (ng/mL)	Min–Max (ng/mL)	Median (ng/mL)
**Whole Group**	163/233 *					
GLIO		71.6%	8.2	1.5	0–114.7	3.9
OTA		74.8%	0.09	0.09	0–0.54	0.08
AFB1		66.2%	0.01	0.01	0–0.06	0.02
AFM1		62.6%	0.12	0.12	0–0.70	0.15
ZEA		51.0%	0.7	1.1	0–6.5	0.8
DON		38.6%	0.7	1.1	0–4.6	0.0
DOM1		27.0%	2.1	4.2	0–19.5	0.0
FB1		44.5%	0.4	1.1	0–12.4	0.0
**Autistic Group**	**114**					
GLIO		69.2%	7.6	13.6	0–82.7	3.5
OTA		70.2%	0.09	0.10	0–0.54	0.08
AFB1		63.2%	0.01	0.01	0–0.06	0.02
AFM1		60.7%	0.12	0.13	0–0.70	0.15
ZEA		50.5%	0.8	1.2	0–6.5	0.8
DON		46.5%	0.9	1.2	0–4.6	0.0
DOM1		32.4%	2.5	4.5	0–19.5	0.0
FB1		43.9%	0.5	1.3	0–12.4	0.0
**Siblings**	**25**					
GLIO		80.0%	11.6	24.7	0–114.7	5.3
OTA		84.0%	0.09	0.07	0–0.27	0.08
AFB1		80.0%	0.01	0.01	0–0.03	0.02
AFM1		84.0%	0.16	0.11	0–0.43	0.15
ZEA		48.0%	0.5	0.7	0–2.8	0.0
DON		16.0%	0.4	1.0	0–3.7	0.0
DOM1		8.0%	0.8	2.8	0–12.0	0.0
FB1		56.0%	0.4	0.5	0–2.0	0.5
**Non-Parental**	**24**					
GLIO		77.3%	7.7	9.8	0–39.7	4.2
OTA		87.5%	0.09	0.05	0–0.19	0.08
AFB1		66.7%	0.01	0.01	0–0.02	0.02
AFM1		54.2%	0.08	0.09	0–0.25	0.15
ZEA		59.1%	0.6	0.8	0–3.17	0.78
DON		25.0%	0.4	0.8	0–2.3	0.0
DOM1		22.7%	1.8	3.6	0–11.4	0.0
FB1		36.4%	0.2	0.4	0–1.5	0.0

* Due to the low amount of samples collected from some children, it was not always possible to carry out all the analyses planned for the study.

**Table 5:** Description of the whole dataset for the mycotoxins analysed in serum samples.

Group	Observations	% Positive	Mean (ng/mL)	Std Dev (ng/mL)	Min–Max (ng/mL)	Median (ng/mL)
**Whole Group**	213/233 *					
GLIO		21.2%	2.3	4.3	0–28.4	0.0
OTA		82.9%	0.36	0.29	0–1.76	0.35
AFB1		22.9%	0.01	0.05	0–0.73	0.00
AFM1		50.2%	0.11	0.18	0–1.91	0.11
ZEA		5.4%	0.1	0.4	0–3.9	0.0
DON		19.5%	1.0	3.6	0–27.9	0.0
DOM1		13.1%	0.3	1.1	0–12.7	0.0
FB1		13.7%	0.2	0.7	0–5.6	0.0
**Autistic Group**	**160**					
GLIO		23.0%	1.8	4.5	0–28.4	0.0
OTA		85.1%	0.39	0.31	0–1.76	0.35
AFB1		24.0%	0.01	0.06	0–0.73	0.00
AFM1		53.2%	0.12	0.20	0–1.91	0.11
ZEA		5.2%	0.1	0.4	0–3.9	0.00
DON		19.5%	1.2	3.9	0–27.9	0.0
DOM1		12.9%	0.3	1.2	0–12.7	0.0
FB1		17.5%	0.3	0.7	0–5.6	0.0
**Siblings**	**35**					
GLIO		14.3%	0.6	1.8	0–5.6	0.0
OTA		77.1%	0.27	0.21	0–0.79	0.29
AFB1		25.7%	0.002	0.00	0–0.01	0.00
AFM1		45.7%	0.07	0.10	0–0.36	0.00
ZEA		8.6%	0.1	0.2	0–1.2	0.0
DON		22.9%	0.5	0.9	0–2.3	0.0
DOM1		17.1%	0.3	0.6	0–1.6	0.0
FB1		2.9%	0.04	0.3	0–1.5	0.0
**Non-Parental**	**18**					
GLIO		18.8%	10.3	33.3	0–1	0.0
OTA		75.0%	0.28	0.2	0–0.61	0.35
AFB1		6.3%	0.00	0.002	0–0.01	0.00
AFM1		31.3%	0.06	0.11	0–0.33	0.00
ZEA		0.0%	0.00	0.0	0–0.0	0.0
DON		12.5%	0.8	2.8	0–11.0	0.0
DOM1		6.3%	0.1	0.3	0–1.4	0.0
FB1		0.0%	0.0	0.0	0–0.0	0.0

* Due to the low amount of samples collected from some children, it was not always possible to carry out all the analyses planned for the study.

**Table 6:** Sub-groups of ASD patients with and without a specific comorbidity.

Sub-Groups with a Specific Co-Morbidity	Sub-Groups without Co-Morbidity
SG1+ Patients with IgG allergenic values >20 mg/L	SG1− Patients without IgG allergenic values ≤20 mg/L
SG2+ Patients with gastrointestinal problems	SG2− Patients without gastrointestinal problems
SG3+ Patients with verbal issue	SG3− Patients without verbal issue
SG4+ Patients with regressive autism	SG3− Patients without regressive autism

**Table 7:** Differences statistically significant in mycotoxin concentration (in urine, _u and serum, _s) between groups and sub-groups.

URINE	GLIO_s	OTA_u	OTA_s	AFB1_u	AFM1_s	FB1_u	DON_u	DOM1_u
Autistic vs. control (all)	-	-	*p* = 0.0141	-	*p* = 0.0072	-	*p* = 0.0141	*p* = 0.0259
Autistic vs. siblings	-	-	*p* = 0.0261	-	*p* = 0.0262	-	*p* = 0.0185	*p* = 0.0259
Autistic vs. siblings paired	-	*p* = 0.0272	*p* = 0.0409	-	-	-	*p* = 0.0305	-
SG1+ (Wheat)	-	-	*p* = 0.0212	-	-	-	-	-
SG1+ (*A. nidulans)*	-	-	-	-	*p* = 0.0457	-	-	-
SG1+ (*A. flavus)*	*p* = 0.0169	-	-	-	-	-	-	-
SG1+ (*C. Albicans)*	-	*p* = 0.0169	-	*p* = 0.0462	-	*p* = 0.0126	-	*p* = 0.0487
SG1+ (*F. Moniliforme)*	-	*p* = 0.0075	-	-	-	-	-	-
SG4+ (regressive pattern)	-	-	-	-	*p* = 0.0385	-	-	

**Table 8:** Correlations between mycotoxin levels and variables (IgGs) in autistic group. Values of ρs for *p* ≤ 0.050 are reported.

**Urine**	**GLIO**	**OTA**	**AFB1**	**FB1**	**DON**	**DOM1**
Gluten	-	-	ρs = 0.2485 *p* = 0.0146	-	-	-
Wheat	-	-	ρs = 0.2188 *p* = 0.0322	-	-	-
*A. clavatus*	-	-	-	ρs = −0.2046 *p* = 0.0455	-	-
*A. flavus*	ρs = −0.2631 *p* = 0.0096	-	-	-	-	-
*A. fumigatus*	-	-	-	-	ρs = −0.2777	-
**Serum**	**GLIO**	**OTA**	**AFB1**	**FB1**	**DON**	**DOM1**
β-lactoglobulin	-	-	-	-	-	-
*A. clavatus*	-	-	ρs = −0.2230 *p* = 0.0290	-	-	-
*A. flavus*	ρs = 0.2258 *p* = 0.0270	-	-	-	-	-
*A. fumigatus*	-	ρs = −0.2466 *p* = 0.0154	-	-	-	-
*A. niger*	-	-	-	-	-	-
*A. orizae*	-	-	ρs = −0.2232 *p* = 0.0288	-	-	-

**Table 9:** Correlations between mycotoxin levels and cytokine/chemokines in autistic group. Values of ρs are reported for all correlations that gave *p* ≤ 0.050.

Cytokines/Chemokines	Serum			Urine	
	OTA	AFM1	DON	AFB1	ZEA
PDGF-BB	-	0.3209	0.2141	-	-
Il-1ra	0.2296	-	-	-	-
Il-4	0.2219	-	-	-	-
Il-5	-	0.2859	0.2519	-	-
Il-7	-	0.3546	0.2484	0.2107	0.2343
Il-12	-	0.2498	-	-	-
Il-13	-	0.2220	-	-	-
IFN-g	0.2195	0.3235	0.2357	-	-
EOT	0.1810	0.3141	-	-	-
TNF-alfa	0.1730	0.2389	-	-	-
RANTES	-	-	0.2348	-	-

**Table 10:** MS/MS conditions for detection of target analytes.

Mycotoxin	Parent [M–H]^+^ (*m*/*z*)	Cone Voltage (*V*)	Quantifier/Qualifier Ion (*m*/*z*)	Collision Energy (eV)	Retention Time (min)
GLIO	327.3	12	263.3/245.3	8/18	6.1
OTA	404.1	20	238.9/341.0	20/20	7.5
AFB1	313.3	30	285.3/128.1	22/22	8.4
AFM1	329.3	30	273.3/259.3	22/22	7.1
ZEA	319.2	10	283.1/301.1	10/10	6.9
DON	297.3	22	249.5/203.5	10/15	4.0
DOM1	281.2	15	108.7/215.1	15/10	4.4
FB1	723.4	50	335.5/353.5	40/30	5.7

**Table 11:** List of variables and their description.

Variables	Description
Location	Bosisio Parini (Lecco, Lombardia, Italy) Ostuni (Brindisi, Puglia, Italy)
Symptom pattern	Verbal/Non-verbal
Questionnaire Information	Age Sex Gastrointestinal problems (Vomit, Diarrhoea, Constipated, Stomach-aches, Painful bowel, Gastroenteritis, Aerophagia, Abdominal bloating, Lack of appetite, Intestinal infections, Intestinal anomalies)
Clinical trial parameters	Food-specific allergological IgG (wheat, gluten, casein, egg yolk, egg white, alfa-lactoalbumin, beta-lactoglobulin) Mould-specific allergological IgG (*Aspergillus fumigatus, A. niger, A. oryzae, A. terreus, A. nidulans, A. flavus, A. clavatus, Candida albicans, Fusarium moniliforme*)
Cytokines	PDGF-BB, Il-1ra, Il-4, Il-5, Il-7, Il-12, Il-13, IFN-g, EOT, TNF-alfa, RANTES
